# The Hospital Officer's Bookshelf

**Published:** 1912-05-04

**Authors:** 


### THE HOSPITAL OFFICER'S BOOKSHELF.

Elements of Anatomy and Physiology: Especially adapted for Nurses. By W. Bernard Secretan, M.B., F.R.C.S. Second edition, revised. Pp. 78. Illustrated. (London: The Scientific Press, Limited. 1912. Price 2s. net.)

Lecturing to nurses is by no means the simple matter it is often thought to be, and the writing of a text-book for junior probationers requires some considerable judgment. Mr. Bernard Secretan has compiled a little book which should prove exceedingly useful as an introduction to anatomy and physiology for the use of first-year nurses and in certain schools. The wording is clear and to the point, while only the most important facts of physiology are dealt with. In view of the fact that for nurses there are in existence many excellent text-books of a wider scope than this one, we feel that the author has done well to err on the side of simplicity. It is easy to suggest the addition of a paragraph here or a note there, but we doubt if the value of the book as a purely elementary text-book would be enhanced thereby. Within its limitations we have no hesitation in recommending this little book as a very useful one for nurses to start on. A second and revised edition has just been published, in which several minor improvements have been made.

Lectures on Midwifery for Midwives. By A. B. Calder, M.B. Second edition. (London: Baillière, Tindall and Cox. 1912. Pp. 262. Price 5s. net.)

In this book the subject of midwifery, in so far as it is necessary for midwives to comprehend it, is dealt with in a series of fifteen lectures. The arrangement is on the whole good, and the language used is well adapted for the audience to whom it is addressed. The teaching is clear and sound, and the pupil midwife who applies it in her practice and in her examinations will emerge successfully from both. There are, naturally, points on which not everyone will agree with the author; fortunately these are not in important matters of principle, but in minor details, where latitude of opinion is admissible. Some of the too glib explanations given of obscure physiological phenomena might well be omitted. Taken all round, however, it gives us pleasure to describe this book as excellently designed for its purpose. The long experience of the author as lecturer and teacher often illumines his pages; and it is not only midwives but their lecturers also who will find the study of this book a profitable undertaking.

National Insurance. By Comyns Carr, Stuart Garnett, and J. H. Taylor. (Macmillan and Co. 6s. net.)

In this book, which commences with a preface specially written by the Chancellor of the Exchequer, the authors describe, simply and broadly, the machinery of the Act, and bring together in a connected form matters which can be gathered only from a careful comparison of the various sections. This connected account is contained in six chapters, forming the first section of the work, and extends to 110 pages of closely printed matter. The first four chapters deal with the Act as it affects (1) the employer, (2) the insured person, (3) those who have to administer its provisions, and (4) the medical profession. In a fifth chapter a full and lucid description is given of sanatorium and maternity benefits, and of the relation of the Public Health Authorities to the Act. The sixth chapter deals in a masterly way with the financial provisions, a clear exposition being given of the necessity for accumulating reserves and of the ingenious manner in which the initial deficiency in reserves is to be liquidated. Unemployment insurance receives attention on a small scale in comparison with health insurance, following in this respect the precedent set in Parliament during the passage of the measure through the House, and giving effect to the undoubted relative importance of the two sections of the Act. Following this descriptive matter the text of the Act and the schedules appended thereto are set out section by section, and numerous comments are made with a view to the interpretation and elucidation of the provisions. This section of the book is so exhaustive that it cannot fail to be of immense service to those who, in any capacity, are engaged in the administration of the Act. A full and well-arranged index is furnished, and tables of cases and statutes cited are also given.

Tuberculin in the Diagnosis and Treatment of Tuberculosis. By W. Camac Wilkinson, M.D., F.R.C.P. (London: James Nisbet and Co. 1912. Price 21s.)

After perusing Dr. Camac Wilkinson's handsome volume, it would be excusable if a layman gathered the impression that the whole of the medical profession of the British Empire is strongly opposed not only to Camac Wilkinson but to tuberculin in any shape or form. It is hardly necessary to mention that this is far from being the attitude of the profession in regard to the use of tuberculin, with which much useful work has already been done by medical men who have not had the advantage of Dr. Wilkinson's tuition. The appearance of a second edition of this work calls for no detailed criticism. We prefer rather to recommend those sufficiently interested to read this lengthy and expensive treatise for themselves. Dr. Wilkinson's methods are his own, and he is prepared to receive and demonstrate to medical men at his Kennington dispensary, where he continues, no doubt, to "prevent innumerable tragedies" and to scare off the family physician who snips off the uvula because his patient had a cough. In addition to the advocacy of tuberculin as the only safe and successful cure for all forms of tuberculosis, the book inveighs abundantly against sanatorium treatment. In this respect the author is probably performing a useful service now that large schemes are being evolved, which will mean the sinking of a great deal of money in sanatorium buildings. In spite of the intense personal note throughout the book, there is much therein which is entitled to careful consideration.

